# Therapieresistente chronische Konjunktivitis

**DOI:** 10.1007/s00105-023-05225-3

**Published:** 2023-09-14

**Authors:** Paul Vorwerk, Linda Dießel, Jens Heichel

**Affiliations:** 1 Klinik und Poliklinik für Augenheilkunde, Universitätsklinikum Halle, Martin-Luther-Universität Halle-Wittenberg, Ernst-Grube-Str. 40, 06120 Halle (Saale), Deutschland; 2 Institut für Pathologie, Universitätsklinikum Halle, Martin-Luther-Universität Halle-Wittenberg, Halle (Saale), Deutschland; 3 Klinik und Poliklinik für Augenheilkunde, Universitätsklinikum Halle, Martin-Luther-Universität Halle-Wittenberg, Halle (Saale), Deutschland

## Anamnese

Ein 25-jähriger Patient stellte sich mit einer seit mehreren Monaten bestehenden Rötung der Augen vor. Neben einem Fremdkörpergefühl klagte der Patient über verstärkte Epiphora. Zudem sei eine langsam größenprogrediente, schmerzlose Neubildung des rechten Unterlides aufgefallen. Anamnestisch waren keine Allergien, Allgemeinerkrankungen oder vorbestehende Augenerkrankungen bekannt. Bestehende Hautverletzungen oder Tierkontakt wurden vom Patienten ebenso wie eine HIV(humanes Immundefizienzvirus)-Infektion oder generelle Immunsuppression verneint. Die Konjunktivitis zeigte sich unter Therapieversuchen mit antibiotischen, steroidalen Augentropfen und antiviralem Augengel sowie Tränenersatzmitteln persistent.

## Klinischer Befund und Diagnostik

**Figure 1:**
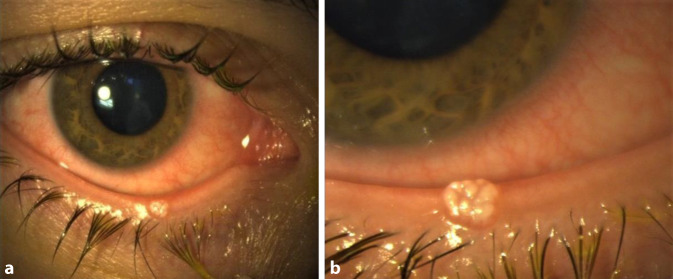

Die Spaltlampenuntersuchung des rechten Auges zeigte ausgeprägte konjunktivale Injektionen bei sonst reizfreiem intraokularem Befund. Am Unterlid präsentierte sich ein mittig gelegener, knotig bis läppchenartig konfigurierter Hauttumor von ca. 1,5 mm Durchmesser und einer Erhabenheit von 0,8 mm (Abb. 1).

Das Ektropionieren ergab keinen Anhalt für einen subtarsalen Fremdkörper. Es bestand eine follikuläre Konjunktivitis. Die fundoskopische Untersuchung in Mydriasis zeigte beidseitig einen unauffälligen und regelrechten Netzhautbefund. Der Visus betrug beidseits unkorrigiert 1,0.

Zur Diagnostik wurde ein Konjunktivalabstrich durchgeführt. Dieser ergab eine Anreicherung von *Bacillus species* sowie *Staphylococcus epidermidis*. Im Verlauf erfolgte die chirurgische Abtragung des Unterlidtumors mit anschließender histopathologischer Begutachtung.

## Histopathologischer Befund

**Figure 2:**
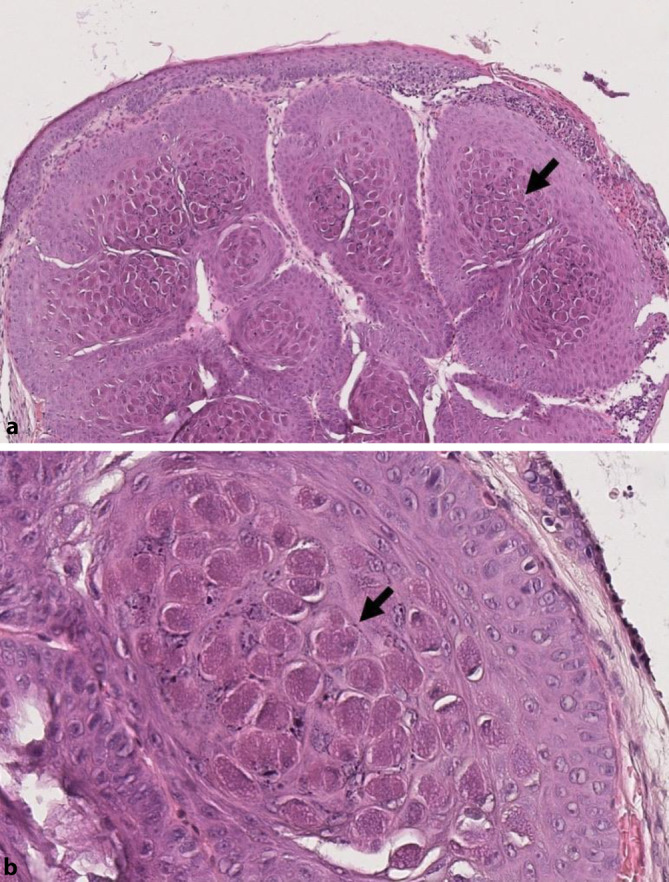

Histologisch zeigte sich ein knotig konfiguriertes Hautexzidat, bedeckt durch dysplasiefreie Epidermis. Diese war abschnittsweise erodiert und granulozytär durchsetzt. Subepithelial lag eine multilobuläre, scharf begrenzte Epithelproliferation vor. Hier imponierten großleibige Zellen mit intrazytoplasmatischen, stark eosinophilen Einschlusskörperchen (Abb. 2). Begleitend waren schollige Keratohyalingranula auffällig, umgebend ein dichtes gemischtes entzündliches Infiltrat.

## Wie lautet Ihre Diagnose?

## Therapie und Verlauf

Nach chirurgischer Abtragung wurde eine topische Therapie mit Neomycinsulfat, Dexamethason und Polymyxin-B-Sulfat für 10 Tage begonnen. Nach Vorliegen der Diagnose wurde die topische Therapie mit Ganciclovir erweitert. Die chronische Konjunktivitis zeigte sich im weiteren Verlauf vollständig rückläufig.

## Diskussion

Mollusken oder sog. Dellwarzen entstehen durch Infektion mit dem Molluscum-contagiosum-Virus, das zu der Familie der Pockenviren gehört [1]. Dabei handelt es sich um behüllte, doppelsträngige DNA(Desoxyribonukleinsäure)-Viren [3].

Obwohl die Infektion in jedem Alter möglich ist, sind Kinder im Alter von 2 bis 10 Jahren am häufigsten betroffen [4]. Ein zweiter Erkrankungsgipfel liegt im frühen Erwachsenenalter zwischen 20 und 25 Jahren [3]. Aufgrund des oft geringen Leidensdruckes erfolgt häufig kein Arztbesuch, sodass genaue Inzidenzwerte der Infektion nicht vorliegen. Sheng et al. untersuchten 2022 in einer retrospektiven Analyse Augenlidtumoren US-amerikanischer pädiatrischer Patienten. Mit 21,9 % war Molluscum contagiosum neben *Verrucae vulgares* (19,0 %) die häufigste pathologisch gesicherte Ursache der Lidtumoren, wobei dabei Fälle mit Chalazion ausgeschlossen wurden [6]. Al-Faky konnte 2012 in 3,2 % der von ihm histopathologisch untersuchten benignen Lidtumoren einer saudi-arabischen Populationsgruppe im Alter zwischen 2 und 87 Jahren eine Molluscum-contagiosum-Infektion als Ursache feststellen [7].

Die Virusübertragung erfolgt überwiegend durch Schmier- und Kontaktinfektion von Mensch zu Mensch, insbesondere bei direktem Hautkontakt. Daher sind v. a. Menschen in warmen, bevölkerungsreichen Umgebungen prädisponiert. Auch durch indirekte Infektionsträger wie Handtücher oder Badewasser oder Autoinokulation kann die weitere Ausbreitung voranschreiten [3].

Klinisch zeigen sich einzelne oder mehrere blasse Papeln mit zentraler Eindellung, die auch konfluieren können. Auf Druck entleert sich virushaltiges infektiöses Zellmaterial. Molluscum-contagiosum-Papeln können den ganzen Körper, insbesondere aber den Rumpf, Extremitäten, Genitalien oder das Gesicht befallen. Lidnahe Läsionen führen oftmals durch Übertritt von kontagiösem Material auf den Tränenfilm zu einer ipsilateralen chronischen follikulären Konjunktivitis [1].

Die Diagnose wird meist klinisch anhand des morphologischen Bildes gestellt, das schlussendlich durch die pathologische Aufarbeitung bestätigt wird. Im histologischen Schnittbild zeigen sich typische intrazytoplasmatische Veränderungen, die sog. Henderson-Patterson-Körperchen [5]. Sie stellen eosinophile Einschlüsse der Keratinozyten im Stratum spinosum und Stratum granulosum der Epidermis dar [1]. Der elektronenmikroskopische Virionennachweis ist ebenfalls möglich.

**Diagnose:** Chronische follikuläre Konjunktivitis bei Molluscum-contagiosum-Infektion

**Figure 3:**
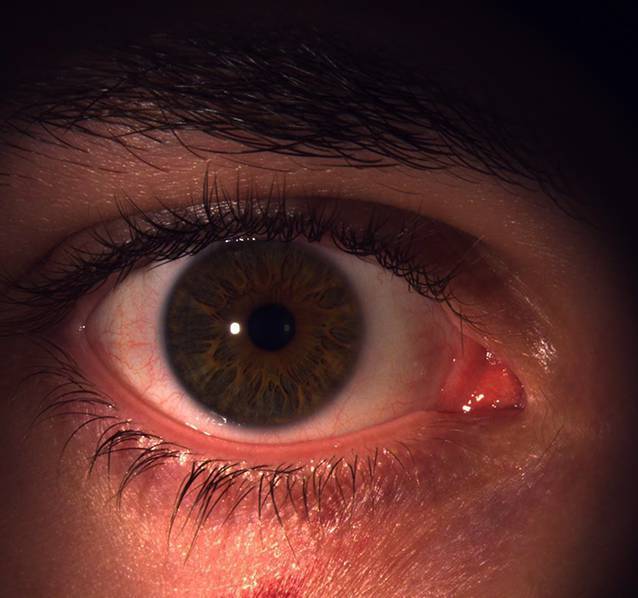

Häufig sind die Hauteffloreszenzen nach 1 bis 2 Jahren selbstlimitierend und bedürfen keiner weiteren Therapie [3]. Bei Persistenz oder beim Auftreten von Komplikationen (wie beispielsweise einer sekundären Konjunktivitis) können die Papeln mittels Exzision, Kauterisation, chemischer Ablation, Kryo- oder Lasertherapie entfernt werden [2]. Andere Indikationen der Exzision können kosmetische Gründe oder die Verhinderung der weiteren Übertragung sein (Abb. 3).

Insbesondere bei Patienten mit atopischer Vorerkrankung oder Immunsuppression (z. B. nach HIV-Infektion oder Chemotherapie) können die Papeln ein schnelleres und größeres Wachstum aufweisen [3] und als Eczema molluscatum disseminieren.

## Fazit für die Praxis

- Bei antibiotikaresistenten Unterlidtumoren ist eine Molluscum-contagiosum-Infektion als Differenzialdiagnose in Betracht zu ziehen.
- Klinisch imponiert eine chronische follikuläre Konjunktivitis.
- Bei Persistenz ist die Exzision/Ablation eine empfohlene Therapieoption.
- Im Falle einer periokulären Manifestation einer Infektion mit Molluscum contagiosum sollte eine interdisziplinäre Betreuung (Dermatologie/Ophthalmologie) eingeleitet werden.
- Mollusca contagiosa der Lidkante können eine atypische Morphe aufweisen, was zu einer verzögerten Diagnosestellung führen kann. Die exzisionale Biopsie hat hier einen hohen Stellenwert.
